# Coadministration of ivermectin and abamectin affects milk pharmacokinetics of the antiparasitic clorsulon in Assaf sheep

**DOI:** 10.3389/fvets.2023.1268658

**Published:** 2023-10-18

**Authors:** Esther Blanco-Paniagua, Laura Álvarez-Fernández, Alicia Millán-García, Guillermo Rivas, Ana I. Álvarez, Gracia Merino

**Affiliations:** ^1^Department of Biomedical Sciences-Physiology, Faculty of Veterinary, University of León, León, Spain; ^2^Instituto de Desarrollo Ganadero y Sanidad Animal (INDEGSAL), University of León, León, Spain

**Keywords:** ABCG2, ivermectin, clorsulon, milk, sheep

## Abstract

In veterinary field, drug exposure during milk production in dairy cattle is considered a major health problem which concerns dairy consumers. The induced expression of the ABC transporter G2 (ABCG2) in the mammary gland during lactation plays a significant role in the active secretion of many compounds into milk. The main objective of this study was to determine the involvement of ABCG2 in the secretion into milk of the antiparasitic clorsulon in sheep as well as the possible effect of the coadministration of model ABCG2 inhibitors such as macrocyclic lactones on this process. Cells transduced with the ovine variant of ABCG2 were used to carry out *in vitro* transepithelial transport assays in which we showed that clorsulon is a substrate of the ovine transporter. In addition, ivermectin and abamectin significantly inhibited clorsulon transport mediated by ovine ABCG2. *In vivo* interactions were studied in Assaf sheep after coadministration of clorsulon (in DMSO, 2 mg/kg, s.c.) with ivermectin (Ivomec^®^, 0.2 mg/kg, s.c.) or abamectin (in DMSO, 0.2 mg/kg, s.c.). After ivermectin and abamectin treatment, no relevant statistically significant differences in plasma levels of clorsulon were reported between the experimental groups since there were no differences in the area under the plasma concentration-curve (AUC) between clorsulon treatment alone and coadministration with macrocyclic lactones. With regard to milk, total amount of clorsulon, as percentage of dose excreted, did not show statistically significant differences when macrocyclic lactones were coadministered. However, the AUC for clorsulon significantly decreased (*p* < 0.05) after coadministration with ivermectin (15.15 ± 3.17 μg h/mL) and abamectin (15.30 ± 3.25 μg h/mL) compared to control group (20.73 ± 4.97 μg h/mL). Moreover, milk parameters such as half-life (*T*_1/2_) and mean residence time (MRT) were significantly lower (*p* < 0.05) after coadministration of macrocyclic lactones. This research shows that the milk pharmacokinetics of clorsulon is affected by the coadministration of ABCG2 inhibitors, reducing drug persistence in milk.

## 1. Introduction

Helminth infections are responsible for the most relevant diseases of livestock globally due to their negative impact on production efficiency in livestock systems. Consequently, control of helminth infections is essential and is mainly based on the use of anthelmintic drugs (1–3). Fascioliasis, a zoonotic disease, causes high economic loses in meat and milk production in livestock throughout the world (4). A number of existing anthelmintic drugs have been used to treat fascioliasis, among them clorsulon (4, 5).

Clorsulon is an antiparasitic drug that belongs to the benzenesulphonamide antiparasitic family used for treatment against adult and mature liver flukes (6). Blocking energy-producing pathways in the fluke is the main mode of action of clorsulon; specifically, it inhibits two enzymes involved in glycolysis of the parasite: 3-phosphate-glyceratekinase and phospho-glyceromutase (6, 7). Regarding its pharmacokinetics, clorsulon is well absorbed after oral administration and eliminated by renal excretion without being metabolized (7, 8). Several previous studies have reported its plasma pharmacokinetics in sheep, goats, cattle and rats (6, 8–10). Commonly, clorsulon is marketed with the macrocyclic lactone ivermectin, combining nematicide and flukicide effects (11, 12). The effectiveness of this combination has been reported in sheep, rats and cattle (13–17).

In veterinary field, potential mechanisms or factors that could modify drug exposure and impact on efficacy as well as on the appearance of drug residues in milk are vital to study. Drug–drug interactions may be one of these factors and must be taken into consideration. Transporter-based interactions in particular have been reported to affect the pharmacokinetics of drugs (17, 18). These interactions are related to coadministration of a drug that is an inhibitor or an inducer of the transporter which may affect the transport of another drug described as a substrate (19). ATP-binding cassette (ABC) transporters are some of the transporters involved in drug–drug interactions and Breast Cancer Resistance Protein (BCRP/ABCG2) is one of the proteins included in this superfamily of transporters, which is the focus of our study.

The ABCG2 transporter extrudes a wide range of drugs from cells due to its localization in the apical membrane of epithelial cells in several tissues such as intestine, kidney, liver, brain and testicles. The main biological function of ABCG2 is to limit toxin accumulation in cells and to modulate of pharmacokinetic processes such as xenobiotic absorption, distribution and elimination (20, 21). Moreover, ABCG2 is expressed in alveolar epithelial cells of the mammary gland during lactation and is one of the main factors involved in active secretion of many compounds into milk (22, 23). Consequently, drugs described as ABCG2 substrates can accumulate in milk, posing a health risk to dairy consumers. Drug–drug interactions mediated by the ABCG2 transporter that lead to the inhibition of ABCG2 affecting drug secretion into milk or plasma availability have been reported (24–30).

Clorsulon has been characterized as an *in vitro* substrate of murine and human variants of ABCG2, and its involvement in the secretion of clorsulon into milk has been reported using ABCG2-knockout mice; furthermore, a drug–drug interaction with the macrocyclic lactone ivermectin has been shown given that its coadministration decreased secretion of clorsulon into milk by ABCG2 in mice (31). However, interaction with the ovine variant of ABCG2 and its potential role in the secretion of clorsulon into milk in sheep is unknown. The purpose of this study was to determine whether clorsulon is an *in vitro* substrate of ovine ABCG2 and to explore its role in the secretion of this flukicide into milk. The effect of macrocyclic lactones, ivermectin and abamectin, on the pharmacokinetics of clorsulon in sheep was also evaluated.

## 2. Materials and methods

### 2.1. Standards and chemicals

Clorsulon and abamectin were purchased from Biosynth Carbosynth (Berkshire, United Kingdom) and albendazol-2 aminosulfone from LGC Standards (Molsheim, France). Ivermectin and Lucifer Yellow were obtained from Sigma-Aldrich (St. Louis, MO, United States). For *in vivo* assays, ivermectin (Ivomec^®^) was purchased from Boehringer Ingelheim (Barcelona, Spain). All the additional chemicals used were reagent grade and were available from commercial suppliers.

### 2.2. Cell cultures

This study employed Madin-Darby Canine Kidney (MDCKII) cells that had been previously transduced with ovine ABCG2. The conditions for culturing have been previously described (32). Briefly, cells were grown in DMEM (Dulbecco’s modified Eagle’s medium) enriched with glutamax (Life Technologies, Inc., Rockville, MD, United States) and supplemented with penicillin (50 units/mL), streptomycin (50 μg/mL), and 10% (v/v) fetal calf serum (MP Biomedicals, Solon, OH, United States) at a temperature of 37°C and 5% of CO_2_. Every 3 to 4 days, cells were trypsinized for subculturing.

### 2.3. Transcellular transport studies

Transcellular transport experiments using ovine ABCG2-transduced cells were conducted following the method previously described (33). Cells were seeded onto microporous polycarbonate membrane filters (3.0 μm pore size, 24 mm diameter; Transwell 3414; Costar, Corning, NY) at a density of 1.0 × 10^6^ per well. The tightness of the monolayer was assessed by measuring its transcellular resistance using Millicell ERS (Millipore Burlington, MA).

Two hours before the beginning, preincubation was carried out replacing medium in both compartments with 2 mL of transport medium [Hanks’ balanced salt solution supplemented with HEPES (25 mM)] with or without the macrocyclic lactones (10 μM ivermectin and 2.5 μM abamectin). The experiment began with the replacement of the medium in either the apical or basal compartment with transport medium containing clorsulon (10 μM), with or without the same concentration of macrocyclic lactones. Aliquots of 100 μL at 1, 2 and 3 h in the opposite compartment were taken, this volume being replaced with fresh medium. At 4 h, 600 μL aliquots were taken in both compartments. Until analysis by high-performance liquid chromatography (HPLC), samples were stored at −20°C. After concluding the experiment, the confluence of the monolayer was evaluated by means of the Lucifer Yellow permeability assay (34). Transport proficiency of these cells was consistently verified through the evaluation of a typical ABCG2 substrate, danofloxacin (25).

The apparent permeability coefficients (*P*_app_) across MDCKII parent and MDCKII oABCG2 cells monolayers in both apical to basal (A-B) (*P*_app_ A-B) and basal to apical (B-A) (*P*_app_ B-A) directions were calculated using following equation: *P*_app_ = (Δ*Q*/Δ*t*)*(1/A*Co), where Δ*Q*/Δ*t* is the rate of corresponding clorsulon appearing in the receiver chamber, which was obtained as the slope of the regression line on the transport time profile of clorsulon across the cell monolayers (from 0 to 4 h); Co is the initial concentration of drug; A is the cell monolayer surface area (4.67 cm^2^). The efflux ratio is the *P*_app_ B-A/*P*_app_ A-B quotient.

### 2.4. Pharmacokinetic experiments with Assaf sheep

Studies with sheep were carried out on the Experimental Farm of the University of León, following institutional guidelines in accordance with European legislation (2010/63/EU). Procedures were also approved by the Animal Care and Use Committee of the University of León ULE-008-2019 (25/09/2019). Eighteen lactating sheep (3–4 months in lactation) of between 77–83 kg were used. Animals were previously dewormed and they had *ad libitum* access to drinking water. Sheep were randomly distributed in three experimental groups. All received a subcutaneous (s.c.) injection of clorsulon at 2 mg/kg (dissolved in DMSO, 80 mg/mL); one group coadministrated with another s.c. injection of ivermectin at 0.2 mg/kg (Ivomec^®^ 1%, Merial, France) and another group with another s.c. injection of abamectin at 0.2 mg/kg (dissolved in DMSO, 8 mg/mL). Blood samples were taken from the jugular vein, while milk samples were obtained after completing milking of the gland before each treatment and at intervals of 1, 2, 4, 6, 8, 10, 12, 24, 30, 48, 72, 96, 120 and 168 h thereafter. Plasma was separated by centrifuging at 1200 g 15 min. Samples were stored at −20°C until analyzed.

### 2.5. High performance liquid chromatography analysis

Samples were analyzed by HPLC under conditions which have been previously described (31). Briefly, the chromatographic system used included a Waters 2695 separation module and a Waters 2998 UV photodiode array detector. Separation was performed on a reversed-phase column (4 mm particle size, 250 × 341 4.6 mm, Max-RP 80Å, 362 Phenomenex^®^, Torrance, CA, United States). The mobile phase consisted of potassium phosphate 0.01 M (pH 7): acetonitrile (75:25). The flow rate was set at 1.20 mL/min, with UV absorbance measured at 225 nm, and the column temperature maintained at 35°C.

*In vitro* samples were injected directly into the HPLC system. *In vivo* samples, milk and plasma, were extracted following a formerly described method (31) with minor modifications. For milk and plasma samples, 10 μL of albendazole-2-aminosulfone (6.25 μg/mL) as internal standard and 200 μL of ethyl acetate were added to each 100 μL. Then, the mixture was vortexed horizontally for 1 min and centrifuged at 8000 g for 10 min at 4°C. The resulting supernatant was collected and subjected to evaporation under N_2_ at 30°C until dryness. To the evaporated samples, 500 μL of hexane and 300 μL of acetonitrile was added, the mixture was again vortexed horizontally for 1 min and centrifuged at 3000 g for 10 min at 4°C. Hexane was removed and the rest was evaporated to dryness under nitrogen stream. After being resuspended in 100 μL of cold methanol, samples were injected into the HPLC system.

Standard samples of clorsulon in the appropriate drug-free matrix were prepared at concentrations of 0.078–10 μg/mL for culture samples, 0.078–2.5 μg/mL for plasma samples, and 0.078–5 μg/mL for milk samples. Correlation coefficients for clorsulon ranged between 0.98–0.99. Precision coefficients of variation were ≤20%, and relative standard deviations (accuracy) values were ≤20%. Determination of the limit of detection (LOD) and limit of quantification (LOQ) was carried out in accordance with the procedure explained by Taverniers et al. (35). LOD was 0.01 μg/mL for cell culture samples and sheep plasma and 0.03 μg/mL for sheep milk. LOQ was 0.02 μg/mL for culture samples, 0.04 μg/mL for sheep plasma and 0.09 μg/mL for sheep milk.

### 2.6. Pharmacokinetic calculations and statistical analyses

Plasma and milk concentrations for each animal were analyzed using PK solution 2.0 computer program (Summit Research Services, Ashland, OH) to calculated pharmacokinetic parameters as previously described (27).

Statistical analysis for significant differences was performed using SPSS Statistics software (v. 26.0; IBM, Armonk, New York, NY, United States). Normal distribution of data was analyzed by the Shapiro–Wilk test. The ANOVA and Kruskal–Wallis tests were applied for normally and not normally distributed data, respectively. A probability of *p* ≤ 0.05 was considered to be statistically significant.

## 3. Results

### 3.1. *In vitro* transport of clorsulon: inhibition by ivermectin and abamectin

**Figure 1:**
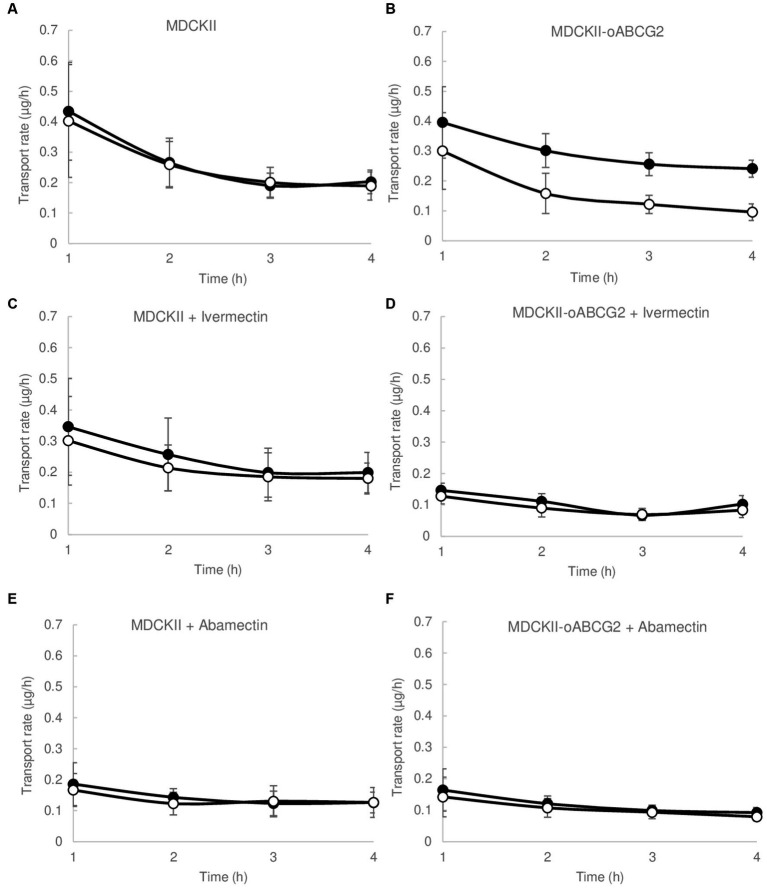
Transepithelial transport assays of clorsulon (10 μM) in MDCKII (parental) cells and its subclones transduced with ovine ABCG2 (oABCG2) **(A,B)**, in presence of ivermectin 10 μM **(C,D)** and abamectin 2.5 μM **(E,F)**. The assay was started with the addition of medium in either the apical or basal compartment with fresh transport medium containing clorsulon with or without ivermectin or abamectin. Results are represented as mean ± SD. Closed circles represent basal to apical (B-A) transport rate and open circles represent apical to basal transport rate (A-B) (*n* ≥ 3).

To determine the *in vitro* role of ovine ABCG2 in transport of clorsulon, MDCKII parental cells and subclone transduced with ovine ABCG2 were used, transepithelial translocation of clorsulon across monolayers on porous membrane filters was measured (Figure 1) and relative efflux ratios were calculated (Table 1).

**Table 1:** Apparent permeability (*P*_app_) values for transepithelial transport of clorsulon (10 μM) across cells monolayers in MDCKII cells and the ovine-ABCG2 transduced cells (oABCG2) with or without ivermectin (10 μM), abamectin (2.5 μM) and Ko143 (1 μM) (*n* ≥ 3).

		BL-AP, ×10^−6^ cm/s (*P*_app_ B-A)	AP-BL, ×10^−6^ cm/s (*P*_app_ A-B)	Efflux ratio *P*_app_ B-A/*P*_app_ A-B
MDCKII	Clorsulon	2.41 ± 0.46	2.27 ± 0.46	1.07 ± 0.06
	Clorsulon + Ivermectin	2.83 ± 0.97	2.61 ± 0.71	1.09 ± 0.17
	Clorsulon + Abamectin	2.29 ± 0.60	2.37 ± 0.93	0.99 ± 0.13
	Clorsulon + Ko143	2.21 ± 0.40	2.15 ± 0.54	1.04 ± 0.08
MDCKII oABCG2	Clorsulon	3.23 ± 0.88	1.13 ± 0.16	2.92 ± 0.93^*^
	Clorsulon + Ivermectin	1.43 ± 0.20	1.22 ± 0.15	1.17 ± 0.11^#^
	Clorsulon + Abamectin	1.67 ± 0.26	1.48 ± 0.30	1.15 ± 0.17^#^
	Clorsulon + Ko143	1.59 ± 0.29	1.50 ± 0.21	1.06 ± 0.09^#^

Results are mean ± SD. ^*^p ≤ 0.05: significant differences from MDCKII cells. ^#^*p* ≤ 0.05: significant differences from MDCKII oABCG2 clorsulon alone treatment.

MDCKII parental cells showed a similar transport in both directions, basolateral and apical (Figure 1A), which is also reflected in the relative efflux ratio, 1.07 ± 0.06. However, translocation in the apical direction was significantly higher (*p* < 0.05) than translocation in the basolateral direction in MDCKII ovine ABCG2 cells (Figure 1B), with a relative efflux ratio significantly higher (*p* < 0.05) in comparison to MDCKII parental cells (Table 1). Specific transport by ABCG2 was checked using a specific inhibitor of ABCG2, Ko143 (36), resulting in a complete inhibition of clorsulon transported by ovine ABCG2-transduced cells. These results reveal that clorsulon is efficiently transported by ovine ABCG2.

We also studied the effect of two macrocyclic lactones, ivermectin and abamectin on the ovine ABCG2-mediated transport of clorsulon and performed transepithelial transport assays in the presence of ivermectin 10 μM (Figures 1C,D) and abamectin 2.5 μM (Figures 1E,F) using MDCKII parental and ovine ABCG2 cells. For ovine ABCG2-transduced cells, clorsulon transport was effectively inhibited in the presence of ivermectin (Figure 1D) and abamectin (Figure 1F), resulting in a similar apical and basolateral translocation, with efflux ratios similar to those in the parental cells (Table 1). These results demonstrate a highly effective *in vitro* inhibitory effect of ivermectin and abamectin on clorsulon transport mediated by ovine ABCG2.

For MDCKII parental cells with ivermectin (Figure 1C) and abamectin (Figure 1E), we showed that clorsulon apical and basolateral translocation were similar. Moreover, relative efflux ratios were comparable to previous efflux ratios obtained in clorsulon alone treatment in MDCKII parental cells (Table 1), thus indicating no effect of these macrocyclic lactones on the parental cells.

### 3.2. Plasma and milk clorsulon pharmacokinetics in sheep

To evaluate the potential *in vivo* drug–drug interactions between macrocyclic lactones and secretion into milk of clorsulon, its coadministration with ivermectin and abamectin was carried out in sheep.

**Figure 2:**
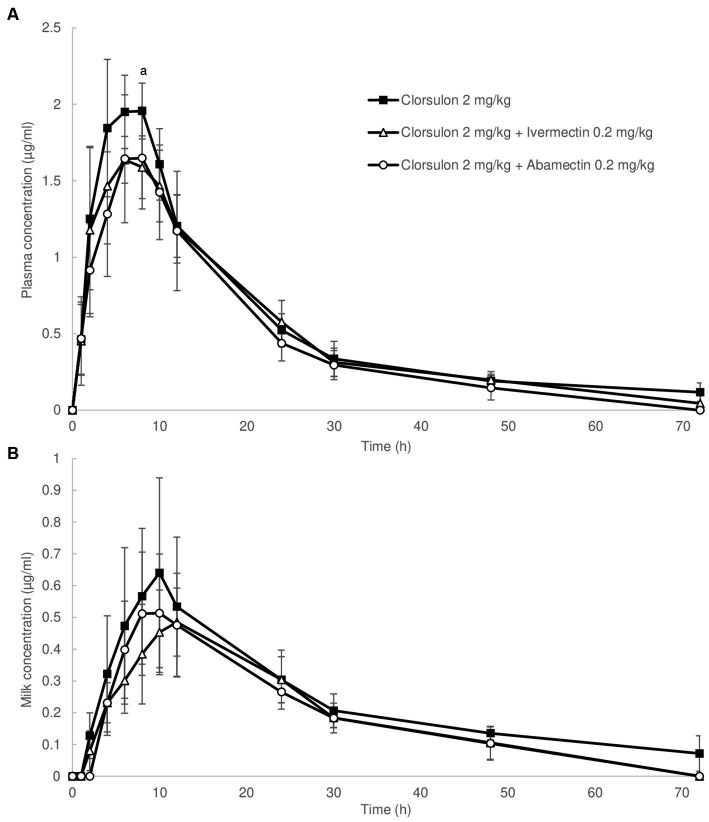
Concentration in plasma **(A)** and milk **(B)** vs. time curves for clorsulon obtained from sheep after s.c. administration of clorsulon at dosage of 2 mg/kg and co-administered with ivermectin at 0.2 mg/kg (s.c.) and abamectin at 0.2 mg/kg (s.c.). Results are represented as mean ± SD (*n* = 6). Lowercase letter (a) represents significant differences (*p* ≤ 0.05) between clorsulon alone dose and ivermectin coadministration.

Mean plasma concentrations are shown in Figure 2A. Clorsulon plasma concentrations were significantly lower (*p* < 0.05) at 8 h (1.59 ± 0.20 μg/mL) after coadministration with ivermectin compared to clorsulon alone treatment (1.95 ± 0.24 μg/mL). Regarding plasma pharmacokinetics parameters (Table 2), *T*_1/2_ and MRT values were lower (*p* < 0.05) after coadministration with abamectin compared to clorsulon administration. However, no significant differences were reported on comparing the AUC for clorsulon between clorsulon alone treatment and coadministration with ivermectin and abamectin. These data indicate that plasma availability was not affected by coadministration of macrocyclic lactones.

**Table 2:** Pharmacokinetic parameters (mean ± SD) for clorsulon in plasma after s.c. administration at 2 mg/kg in sheep co-administrated with ivermectin (0.2 mg/kg, s.c.) and abamectin (0.2 mg/kg, s.c.) (*n* = 6).

Parameters^a^	Clorsulon	Clorsulon + ivermectin	Clorsulon + abamectin
*C*_max_ (μg/mL)	2.13 ± 0.28	1.72 ± 0.19	1.70 ± 0.40
*T*_max_ (h)	6.67 ± 1.93	6.00 ± 1.79	7.67 ± 0.82
*T*_1/2_ (h)	18.97 ± 2.38	15.74 ± 3.83	12.34 ± 2.65^*^
MRT (h)	25.00 ± 3.97	22.85 ± 5.20	18.82 ± 2.96^*^
AUC _(0–∞)_ (μg h/mL)	43.27 ± 6.40	38.77 ± 6.10	33.68 ± 9.07

*p ≤ 0.05, significant differences from clorsulon alone treatment.

a *C*_max_, maximum concentration; *T*_max_, time to maximum concentration; *T*_1/2_, half-life; MRT, mean residence time; AUC, area under the curve.

Regarding milk, mean concentrations (Figure 2B) and pharmacokinetic parameters of clorsulon (Table 3) were calculated. No differences were reported in mean milk concentrations between clorsulon alone administration and coadministration with ivermectin or abamectin (Figure 2B). Nevertheless, the milk AUC for clorsulon was around 25% lower (*p* < 0.05) after coadministration with ivermectin and abamectin compared to clorsulon alone treatment (Table 3). No differences were reported for the AUC milk-to-plasma values or total amount of clorsulon as percentage of dose excreted between clorsulon alone treatment and ivermectin or abamectin coadministration (Table 3). With regard to pharmacokinetics parameters, the *T*_1/2_ values were lower (*p* < 0.05) after coadministration with ivermectin and abamectin compared to clorsulon administration. Furthermore, the MRT showed a reduction (*p* < 0.05) in milk values after coadministration of ivermectin and abamectin compared to clorsulon alone treatment.

**Table 3:** Pharmacokinetic parameters (mean ± SD) for clorsulon in milk after s.c. administration at 2 mg/kg in sheep co-administered with ivermectin (0.2 mg/kg, s.c.) and abamectin (0.2 mg/kg, s.c.) (*n* = 6).

Parameters^a^	Clorsulon	Clorsulon + ivermectin	Clorsulon + abamectin
*C*_max_ (μg/mL)	0.65 ± 0.29	0.52 ± 0.12	0.57 ± 0.19
*T*_max_ (h)	10.00 ± 1.26	11.00 ± 1.67	10.00 ± 1.79
*T*_1/2_ (h)	25.88 ± 3.72	17.44 ± 5.51	17.90 ± 4.56
MRT (h)	40.77 ± 6.15	30.45 ± 7.83^*^	30.27 ± 6.91^*^
AUC _(0–∞)_ (μg h/mL)	20.73 ± 4.97	15.15 ± 3.17^*^	15.30 ± 3.25^*^
AUC milk/AUC plasma	0.49 ± 0.14	0.39 ± 0.03	0.47 ± 0.11
Dose excreted (%) in milk (72 h)	0.86 ± 0.28	0.75 ± 0.40	0.59 ± 0.18

^*^p ≤ 0.05, significant differences from clorsulon alone treatment.

a *C*_max_, maximum concentration; *T*_max_, time to maximum concentration; *T*_1/2_, half-life; MRT, mean residence time; AUC, area under the curve.

These results indicate that milk pharmacokinetics of clorsulon is modified following its simultaneous administration with the macrocyclic lactones ivermectin and abamectin in sheep. In addition, a reduction in the persistence of clorsulon in milk was reported.

## 4. Discussion

Animal helminthic infections have an important unwanted impact not only on livestock production, but also on animal welfare, the environment and human health. Prevention or treatment of these diseases using chemotherapeutics is essential for their control. Nevertheless, there are undesired side-effects related to anthelmintic use such as anthelmintic resistance development and anthelmintic residues in the environment and animal-derived products (3). Moreover, antiparasitic combinations are often used to enhance the spectrum of activity. For example, clorsulon with the macrocyclic lactone ivermectin is a marketed combination used in veterinary medicine as a broad-spectrum anthelmintic formulation thanks to the association of a nematicide and a flukicide (11, 12). However, the administration of different anthelmintic drugs in combination can lead to unpredictable drug–drug interactions that must be considered. In particular, transporter-based interactions may affect plasma and milk pharmacokinetics parameters of drugs (19, 23, 37, 38).

Recently, we showed that clorsulon was efficiently transported *in vitro* by murine ABCG2 and human ABCG2, and also that ivermectin inhibited murine and human ABCG2-mediated transport of clorsulon. Moreover, we reported a reduction in milk levels of clorsulon after coadministration of ivermectin in mice (31). In accordance with these outcomes, in the present study we show that clorsulon is an *in vitro* substrate for ovine ABCG2 and that there is an important effect of two macrocyclic lactones, ivermectin and abamectin, as model ABCG2 inhibitors, on the transfer of clorsulon into sheep milk.

*In vitro* transcellular transport assays using MDCKII cells transduced with ovine variant of ABCG2 showed that clorsulon is efficiently transported by ovine ABGC2 with an efflux transport ratio of 2.92 ± 0.93 (Table 1, Figure 1B). Similar ratios were previously obtained in murine ABCG2 (2.20 ± 0.13) and human ABCG2 (1.63 ± 0.17) transduced cells lines (31). Beforehand, different compounds were described as substrates of ovine ABCG2 such as antibiotics and non-steroidal anti-inflammatories (28, 30, 32, 39).

We also demonstrate an efficient *in vitro* inhibition of ABCG2-mediated transport of clorsulon in ovine variant by the macrocyclic lactones ivermectin and abamectin (Table 1). Ivermectin and abamectin are avermectin compounds known for their anthelmintic and insecticidal effects (40). Both of these have been described as inhibitors of the ABC transporter P-glycoprotein (41). Nevertheless, only ivermectin has been previously identified as an *in vitro* inhibitor of ABCG2, with an inhibition potency value of 45.9% at 25 μM in ovine ABCG2-transduced cells (32, 42). Furthermore, this inhibitory effect was confirmed using transport assays with well-known ABCG2 substrates such as danofloxacin (25), albendazole sulphoxide (43) or novel substrates like meloxicam (30). Apart from ivermectin, other avermectins have been reported to have inhibitory effects on ABCG2 such as doramectin (32), eprinomectin (29) and selamectin (42). However, the interaction between abamectin and ABCG2 has not yet been described. Due to its similar structure with the rest of the avermectins, its hydrophobic properties, previously related as a common chemical feature of inhibitors of ABCG2 (44), as well as its current importance in the agriculture field (40), it was interesting to address the potential role of abamectin as an inhibitor of ovine ABCG2.

Following the meaningful inhibition obtained in the *in vitro* assays, the extent of *in vivo* ABCG2-mediated drug–drug interaction involving macrocyclic lactones and the ovine ABCG2 substrate clorsulon was determined carrying out the coadministration of clorsulon with ivermectin and abamectin in sheep. Clorsulon was administrated at the recommended dose of 2 mg/kg by the s.c. route, as is commonly used in cattle (6), and ivermectin and abamectin were tested at doses based on the recommended dose rate (45).

Plasma concentrations of clorsulon (Figure 2A) were in the same range as previous pharmacokinetics studies carried out in cattle in which after s.c. administration of 2 mg/kg of clorsulon, the maximum plasma concentration was 2.5 μg/mL at 6 h (6). We showed that plasma concentrations were lower at 8 h after coadministration with ivermectin. Differences in *T*_1/2_ and MRT after coadministration with abamectin were reported with lower values compared to clorsulon alone administration. Despite these differences in clorsulon plasma concentrations at certain single time points and pharmacokinetics parameters, no significant differences between AUC values in clorsulon alone treatment compared to coadministration of macrocyclic lactones were reported, although these differences tended to be lower after the coadministrations (Table 2). Lack of differences in clorsulon plasma availability in sheep after macrocyclic lactone coadministration is in agreement with the lack of differences in clorsulon plasma levels in mice after coadministration with ivermectin (31). In particular, there were no differences in plasma concentrations of clorsulon between wild-type and Abcg2^−/−^ mice in the treatment either with clorsulon alone or with the combination of clorsulon and ivermectin. Even when comparing treatments with or without ivermectin in both types of mice, no differences in plasma concentrations of clorsulon were reported. Although our *in vitro* results evidently showed that clorsulon is a substrate of ovine ABCG2 and that this transporter can have an influence on the plasma disposition of its substrates (20), it is important to consider that other factors, including the potential involvement of other transporters *in vivo*, might conceal the effect of ABCG2 on the systemic disposition of clorsulon.

Focusing on milk pharmacokinetics of clorsulon, in our study the effect of ivermectin and abamectin on clorsulon pharmacokinetics in milk was also studied in Assaf sheep (Figure 2B and Table 3). Milk AUC values decreased following coadministration of ivermectin and abamectin compared to treatment with clorsulon alone (Table 3). Moreover, the values of the parameters *T*_1/2_ and MRT in milk were also lower after coadministration of macrocyclic lactones compared to clorsulon alone treatment (Table 3), showing a reduction in the persistence of clorsulon in milk. Previous studies reported a reduction in milk levels of ABCG2 substrates such as danofloxacin (25) or meloxicam (30) after coadministration of ivermectin in sheep, without any effect in plasma levels. In addition, the coadministration of different ABCG2 inhibitors in sheep, such as triclabendazole metabolites (28) or soy isoflavones (26) were also reported to affect milk pharmacokinetics parameters of moxidectin and danofloxacin, respectively.

Our results show no significant differences in AUC milk-to-plasma values of clorsulon between experimental groups. We cannot rule out the possibility that the coadministration of macrocyclic lactones affected systemic exposure of clorsulon by some unknown mechanism since reduced AUC in plasma was also shown although not statistically significant, probably due to high interindividual variability. Consequently, reduction in milk AUC might be a consequence of this potential plasma reduction. In any case, these results show that coadministration of macrocyclic lactones such as model ABCG2 inhibitors causes a reduction in the persistence of clorsulon in milk, limiting potential exposure of the offspring and consumers of dairy products to this xenobiotic and enhancing the understanding of the potential factors that could affect the pharmacokinetics of contaminants in milk.

## 5. Conclusion

Our study defined clorsulon as an *in vitro* substrate of ovine ABCG2 and showed the *in vitro* inhibitory effect of ivermectin and abamectin on clorsulon transport mediated by ovine ABCG2. In sheep, the coadministration of ABCG2 inhibitors such as macrocyclic lactones affected milk pharmacokinetics of clorsulon.

## Data availability statement

The raw data supporting the conclusions of this article will be made available by the authors, without undue reservation.

## Ethics statement

The animal studies were approved by Animal Care and Use Committee of the University of León (ULE-008-2019). The studies were conducted in accordance with the local legislation and institutional requirements. Written informed consent was obtained from the owners for the participation of their animals in this study.

## Author contributions

EB-P: Conceptualization, Data curation, Formal analysis, Investigation, Methodology, Writing – original draft. LÁ-F: Data curation, Formal analysis, Methodology, Investigation, Writing – review & editing. AM-G: Investigation, Writing – review & editing. GR: Methodology, Writing – review & editing. AÁ: Methodology, Validation, Conceptualization, Funding acquisition, Supervision, Writing – review & editing. GM: Methodology, Conceptualization, Funding acquisition, Project administration, Resources, Validation, Writing – review & editing.

## Abbreviations

ABC: ATP-binding cassette
AP-BL: Apical to basal transport
AUC: Area under concentration-time curves
BCRP/ABCG2: Breast cancer resistance protein/ATP-binding cassette transporter G2
BL-AP: Basal to apical transport
*C*_max_: Maximum peak concentration
HPLC: High-performance liquid chromatography
LOD: Limit of detection
LOQ: Limit of quantification
MDCKII: Madin-Darby canine kidney
MRT: Mean residence time
s.c.: Subcutaneous
*T*_max_: Time to maximum concentration
*T*_1/2_: Half-life

## References

[1] Duthaler U Smith TA Keiser J. *In vivo* and *in vitro* sensitivity of *Fasciola hepatica* to triclabendazole combined with artesunate, artemether, or OZ78. Antimicrob Agents Chemother. (2010) 54:4596–604. doi: 10.1128/AAC.00828-10, PMID: 20733042 PMC2976137

[2] Statham JME. Control of liver fluke: an emerging issue in terms of veterinary residues. Vet Rec. (2015) 177:519–21. doi: 10.1136/vr.h6287, PMID: 26589987

[3] Vercruysse J Charlier J Van Dijk J Morgan ER Geary T von Samson-Himmelstjerna G. Control of helminth ruminant infections by 2030. Parasitology. (2018) 145:1655–64. doi: 10.1017/S003118201700227X, PMID: 29415781

[4] Siles-Lucas M Becerro-Recio D Serrat J González-Miguel J. Fascioliasis and fasciolopsiasis: current knowledge and future trends. Res Vet Sci. (2021) 134:27–35. doi: 10.1016/j.rvsc.2020.10.011 33278757

[5] Martínez-Valladares M Cordero-Pérez C Rojo-Vázquez FA. Efficacy of an anthelmintic combination in sheep infected with *Fasciola hepatica* resistant to albendazole and clorsulon. Exp Parasitol. (2014) 136:59–62. doi: 10.1016/j.exppara.2013.10.010, PMID: 24211419

[6] European Medicines Agency. Committee for medicinal products for veterinary use. European public MRL assessment report (EPMAR). EMEA/CVMP/358525/2008. (2008). Available at: https://www.ema.europa.eu/en/documents/mrl-report/clorsulon-cattle-european-public-mrl-assessment-report-epmar-committee-veterinary-medicinal-products_en.pdf

[7] Richter D Richter J Grüner B Kranz K Franz J Kern P. *In vitro* efficacy of triclabendazole and clorsulon against the larval stage of echinococcus multilocularis. Parasitol Res. (2013) 112:1655–60. doi: 10.1007/s00436-013-3321-7, PMID: 23455934

[8] Sundlof SF Whitlock TW. Clorsulon pharmacokinetics in sheep and goats following oral and intravenous administration. J Vet Pharmacol Ther. (1992) 15:282–91. doi: 10.1111/j.1365-2885.1992.tb01018.x, PMID: 1433492

[9] Schulman MD Valentino D Cifelli S Lang R Ostlind DA. A pharmacokinetic basis for the efficacy of 4-amino-6-trichloroethenyl-1,3-benzenedisulfonamide against *Fasciola hepatica* in the rat. J Parasitol. (1979) 65:555–61. doi: 10.2307/3280320, PMID: 512752

[10] Schulman MD Valentino D Cifelli S Ostlind DA. Dose-dependent pharmacokinetics and efficacy of MK-401 against old, and young-mature infections of *Fasciola hepatica* in the rat. J Parasitol. (1982) 68:603–8. doi: 10.2307/3280917, PMID: 7119988

[11] Dupuy J Alvinerie M Ménez C Lespine A. Interaction of anthelmintic drugs with P-glycoprotein in recombinant LLC-PK1-mdr1a cells. Chem Biol Interact. (2010) 186:280–6. doi: 10.1016/j.cbi.2010.05.013, PMID: 20513441

[12] Meaney M Fairweather I Brennan GP McDowell LSL Forbes AB. *Fasciola hepatica*: effects of the fasciolicide clorsulon *in vitro* and *in vivo* on the tegumental surface, and a comparison of the effects on young- and old-mature flukes. Parasitol Res. (2003) 91:238–50. doi: 10.1007/s00436-003-0863-0, PMID: 12937959

[13] Heredia R Aguilar E Romero C Bautista L Mendoza G. Evaluation of five treatments to control intestinal parasites in sheep in Ayapango, state of Mexico. Vet World. (2016) 9:1233–7. doi: 10.14202/vetworld.2016.1233-1237, PMID: 27956774 PMC5146303

[14] Sibille P Calléja C Carreras F Bigot K Galtier P Boulard C. *Fasciola hepatica*: influence of gender and liver biotransformations on flukicide treatment efficacy of rats infested and cured with either clorsulon/ivermectin or triclabendazole. Exp Parasitol. (2000) 94:227–37. doi: 10.1006/expr.2000.4501 10831390

[15] Geurden T Bartram D Van Brussel L Bo L Scott-Baird E Rugg D. Evaluation of the comparative efficacy of a moxidectin plus triclabendazole pour-on solution against adult and immature liver fluke, *Fasciola hepatica*, in cattle. Vet Parasitol. (2012) 189:227–32. doi: 10.1016/j.vetpar.2012.04.019, PMID: 22579500

[16] Ibarra-Velarde F Vera-Montenegro Y Nájera-Fuentes R Sánchez-Albarran A. Efficacy of combined chemotherapy against gastrointestinal nematodes and *Fasciola hepatica* in cattle. Vet Parasitol. (2001) 99:199–204. doi: 10.1016/S0304-4017(01)00460-5 11502367

[17] Marquez B Van Bambeke F. ABC multidrug transporters: target for modulation of drug pharmacokinetics and drug–drug interactions. Curr Drug Targets. (2011) 12:600–20. doi: 10.2174/138945011795378504, PMID: 21039335

[18] Hillgren KM Keppler D Zur AA Giacomini KM Stieger B Cass CE. Emerging transporters of clinical importance: an update from the International Transporter Consortium. Clin Pharmacol Ther. (2013) 94:52–63. doi: 10.1038/clpt.2013.74, PMID: 23588305

[19] Zhang L Reynolds KS Zhao P Huang S-M. Drug interactions evaluation: an integrated part of risk assessment of therapeutics. Toxicol Appl Pharmacol. (2010) 243:134–45. doi: 10.1016/j.taap.2009.12.016, PMID: 20045016

[20] Vlaming MLH Lagas JS Schinkel AH. Physiological and pharmacological roles of ABCG2 (BCRP): recent findings in Abcg2 knockout mice. Adv Drug Deliv Rev. (2009) 61:14–25. doi: 10.1016/j.addr.2008.08.007, PMID: 19118589

[21] Meyer zu Schwabedissen HE Kroemer HK. *In vitro* and *in vivo* evidence for the importance of breast cancer resistance protein transporters (BCRP/MXR/ABCP/ABCG2) Handb Exp Pharmacol. (2011). 201:325–71. doi: 10.1007/978-3-642-14541-4_9 21103975

[22] Jonker JW Merino G Musters S van Herwaarden AE Bolscher E Wagenaar E. The breast cancer resistance protein BCRP (ABCG2) concentrates drugs and carcinogenic xenotoxins into milk. Nat Med. (2005) 11:127–9. doi: 10.1038/nm1186, PMID: 15685169

[23] Garcia-Lino AM Álvarez-Fernández I Blanco-Paniagua E Merino G Álvarez AI. Transporters in the mammary gland—contribution to presence of nutrients and drugs into milk. Nutrients. (2019) 11:2372. doi: 10.3390/nu11102372, PMID: 31590349 PMC6836069

[24] Pulido MM Molina AJ Merino G Mendoza G Prieto JG Alvarez AI. Interaction of enrofloxacin with breast cancer resistance protein (BCRP/ABCG2): influence of flavonoids and role in milk secretion in sheep. J Vet Pharmacol Ther. (2006) 29:279–87. doi: 10.1111/j.1365-2885.2006.00744.x, PMID: 16846465

[25] Real R Egido E Pérez M González-Lobato L Barrera B Prieto JG. Involvement of breast cancer resistance protein (BCRP/ABCG2) in the secretion of danofloxacin into milk: interaction with ivermectin. J Vet Pharmacol Ther. (2011) 34:313–21. doi: 10.1111/j.1365-2885.2010.01241.x 20950350

[26] Perez M Otero JA Barrera B Prieto JG Merino G Alvarez AI. Inhibition of ABCG2/BCRP transporter by soy isoflavones genistein and daidzein: effect on plasma and milk levels of danofloxacin in sheep. Vet J. (2013) 196:203–8. doi: 10.1016/j.tvjl.2012.09.012 23083838

[27] Otero JA García-Mateos D Alvarez-Fernández I García-Villalba R Espín JC Álvarez AI. Flaxseed-enriched diets change milk concentration of the antimicrobial danofloxacin in sheep. BMC Vet Res. (2018) 14:14. doi: 10.1186/s12917-018-1341-3, PMID: 29334949 PMC5769330

[28] Barrera B González-Lobato L Otero JA Real R Prieto JG Álvarez AI. Effects of triclabendazole on secretion of danofloxacin and moxidectin into the milk of sheep: role of triclabendazole metabolites as inhibitors of the ruminant ABCG2 transporter. Vet J. (2013) 198:429–36. doi: 10.1016/j.tvjl.2013.07.033 23981352

[29] Garcia-Lino AM Garcia-Mateos D Alvarez-Fernandez I Blanco-Paniagua E Medina JM Merino G. Role of eprinomectin as inhibitor of the ruminant ABCG2 transporter: effects on plasma distribution of danofloxacin and meloxicam in sheep. Res Vet Sci. (2021) 136:478–83. doi: 10.1016/j.rvsc.2021.03.026, PMID: 33838457

[30] Blanco-Paniagua E Garcia-Lino AM Alvarez-Fernández L Alvarez AI Merino G. Ivermectin inhibits ovine ABCG2-mediated *in vitro* transport of meloxicam and reduces its secretion into milk in sheep. Res Vet Sci. (2022) 153:88–91. doi: 10.1016/j.rvsc.2022.10.019 36327623

[31] Blanco-Paniagua E Álvarez-Fernández L Rodríguez-Alonso A Millán-Garcia A Álvarez AI Merino G. Role of the Abcg2 transporter in secretion into milk of the anthelmintic clorsulon: interaction with ivermectin. Antimicrob Agents Chemother. (2023) 67:e0009523. doi: 10.1128/aac.00095-23, PMID: 37078871 PMC10190675

[32] González-Lobato L Real R Herrero D de la Fuente A Prieto JG Marqués MM. Novel *in vitro* systems for prediction of veterinary drug residues in ovine milk and dairy products. Food Addit Contam A. (2014) 31:1026–37. doi: 10.1080/19440049.2014.908261 24679113

[33] Merino G Jonker JW Wagenaar E van Herwaarden AE Schinkel AH. The breast cancer resistance protein (BCRP/ABCG2) affects pharmacokinetics, hepatobiliary excretion, and milk secretion of the antibiotic nitrofurantoin. Mol Pharmacol. (2005) 67:1758–64. doi: 10.1124/mol.104.010439, PMID: 15709111

[34] Mahnke H Ballent M Baumann S Imperiale F von Bergen M Lanusse C. The ABCG2 efflux transporter in the mammary gland mediates veterinary drug secretion across the blood-milk barrier into milk of dairy cows. Drug Metab Dispos. (2016) 44:700–8. doi: 10.1124/dmd.115.068940, PMID: 26956640

[35] Taverniers I De Loose M Van Bockstaele E. Trends in quality in the analytical laboratory: II. Analytical method validation and quality assurance. Trends Anal Chem. (2004) 23:535–52. doi: 10.1016/j.trac.2004.04.001

[36] Allen JD van Loevezijn A Lakhai JM van der Valk M van Tellingen O Reid G. Potent and specific inhibition of the breast cancer resistance protein multidrug transporter *in vitro* and in mouse intestine by a novel analogue of fumitremorgin C. Mol Cancer Ther. (2002) 1:417–25. Available at: https://aacrjournals.org/mct/article/1/6/417/233728/Potent-and-Specific-Inhibition-of-the-Breast 12477054

[37] Neodo A Schulz JD Huwyler J Keiser J. *In vitro* and *in vivo* drug-drug interaction study of the effects of ivermectin and oxantel pamoate on tribendimidine. Antimicrob Agents Chemother. (2019) 63:e00762–18. doi: 10.1128/AAC.00762-18 30323047 PMC6325189

[38] Ballent M Canton C Dominguez P Bernat G Lanusse C Virkel G. Pharmacokinetic-pharmacodynamic assessment of the ivermectin and abamectin nematodicidal interaction in cattle. Vet Parasitol. (2020) 279:109010. doi: 10.1016/j.vetpar.2019.109010, PMID: 32035291

[39] Wassermann L Halwachs S Lindner S Honscha KU Honscha W. Determination of functional ABCG2 activity and assessment of drug–ABCG2 interactions in dairy animals using a novel MDCKII *in vitro* model. J Pharm Sci. (2013) 102:772–84. doi: 10.1002/jps.23399, PMID: 23192864

[40] El-Saber Batiha G Alqahtani A Ilesanmi OB Saati AA El-Mleeh A Hetta HF. Avermectin derivatives, pharmacokinetics, therapeutic and toxic dosages, mechanism of action, and their biological effects. Pharmaceuticals. (2020) 13:196. doi: 10.3390/ph13080196 32824399 PMC7464486

[41] Lespine A Martin S Dupuy J Roulet A Pineau T Orlowski S. Interaction of macrocyclic lactones with P-glycoprotein: structure-affinity relationship. Eur J Pharm Sci. (2007) 30:84–94. doi: 10.1016/j.ejps.2006.10.004, PMID: 17134887

[42] Merino G Real R Baro MF Gonzalez-Lobato L Prieto JG Alvarez AI. Natural allelic variants of bovine ATP-binding cassette transporter ABCG2: increased activity of the Ser581 variant and development of tools for the discovery of new ABCG2 inhibitors. Drug Metab Dispos. (2009) 37:5–9. doi: 10.1124/dmd.108.022715, PMID: 18824523

[43] Muenster U Grieshop B Ickenroth K Gnoth MJ. Characterization of substrates and inhibitors for the *in vitro* assessment of Bcrp mediated drug–drug interactions. Pharm Res. (2008) 25:2320–6. doi: 10.1007/s11095-008-9632-1 18523872

[44] Egido E Müller R Li-Blatter X Merino G Seelig A. Predicting activators and inhibitors of the breast cancer resistance protein (ABCG2) and P-glycoprotein (ABCB1) based on mechanistic considerations. Mol Pharm. (2015) 12:4026–37. doi: 10.1021/acs.molpharmaceut.5b00463, PMID: 26372856

[45] European Medicines Agency. European public MRL assessment report (EPMAR). clorsulon (bovine milk)—after the provisional maximum residue limit (MRL). EMA/CVMP/752339/2013 (2014) Available at: https://www.ema.europa.eu/en/documents/mrl-report/clorsulon-bovine-milk-european-public-maximum-residue-limit-assessment-report-epmar-cvmp_en.pdf

